# Inhibitory Effects of *Ecklonia cava* Extract on High Glucose-Induced Hepatic Stellate Cell Activation

**DOI:** 10.3390/md9122793

**Published:** 2011-12-20

**Authors:** Kumiko Yokogawa, Isao Matsui-Yuasa, Akiko Tamura, Masaki Terada, Akiko Kojima-Yuasa

**Affiliations:** 1 Department of Food and Human Health Sciences, Graduate School of Human Life Science, Osaka City University, 3-3-138 Sugimoto, Sumiyoshi-ku, Osaka 558-8585, Japan; Email: kumi_sr_93528@yahoo.co.jp (K.Y.); yuasa-i@hotmail.co.jp (I.M.-Y.); azsxd_22@yahoo.co.jp (A.T.); 2 Faculty of Education, Wakayama University, 930 Sakaedani, Wakayama 640-8510, Japan; 3 JP Renew Distributors, LLC., 1906 Lombard Street, San Francisco, CA 94123, USA; Email: macterad@bc4.so-net.ne.jp

**Keywords:** type I collagen, *Ecklonia cava*, reactive oxygen species, transforming growth factor-β, high glucose, hepatic stellate cells

## Abstract

Nonalcoholic steatohepatitis (NASH) is a disease closely associated with obesity and diabetes. A prevalence of type 2 diabetes and a high body mass index in cryptogenic cirrhosis may imply that obesity leads to cirrhosis. Here, we examined the effects of an extract of *Ecklonia cava*, a brown algae, on the activation of high glucose-induced hepatic stellate cells (HSCs), key players in hepatic fibrosis. Isolated HSCs were incubated with or without a high glucose concentration. *Ecklonia cava* extract (ECE) was added to the culture simultaneously with the high glucose. Treatment with high glucose stimulated expression of type I collagen and α*-*smooth muscle actin, which are markers of activation in HSCs, in a dose-dependent manner. The activation of high glucose-treated HSCs was suppressed by the ECE. An increase in the formation of intracellular reactive oxygen species (ROS) and a decrease in intracellular glutathione levels were observed soon after treatment with high glucose, and these changes were suppressed by the simultaneous addition of ECE. High glucose levels stimulated the secretion of bioactive transforming growth factor-β (TGF-β) from the cells, and the stimulation was also suppressed by treating the HSCs with ECE. These results suggest that the suppression of high glucose-induced HSC activation by ECE is mediated through the inhibition of ROS and/or GSH and the downregulation of TGF-β secretion. ECE is useful for preventing the development of diabetic liver fibrosis.

## 1. Introduction

A prevalence of type 2 diabetes mellitus and a high body mass index in cryptogenic cirrhosis may imply that obesity leads to cirrhosis [1,2]. In addition, hyperglycemia and hyperinsulinemia may accelerate liver fibrosis and cirrhosis [3]. It has also been reported that advanced glycation end products (the final reaction products of proteins with sugars) induce reactive oxygen species (ROS) generation and intensify the proliferation and activation of hepatic stellate cells (HSCs), key players in hepatic fibrosis [4].

It has been shown that an induction of collagen gene expression in renal fibroblasts by high glucose levels is one of the mechanisms of diabetic nephropathy. Moreover, hyperglycemia has been shown to be functionally related to fibrogenesis in a model of diabetic nephropathy in which the progressive accumulation of extracellular matrix (ECM) components was observed in the glomerular mesangium and tubulointerstitium [5]. HSCs are a major source of ECM, and during fibrogenesis, they undergo an activation process that is characterized by increased proliferation and collagen synthesis. Therefore, it is important to establish an *in vitro* model of diabetic fibrosis with HSCs to clarify the mechanism of high glucose-induced fibrogenesis in nonalcoholic steatohepatitis (NASH). In this regard, Sugimoto *et al*. have shown that high glucose concentrations (450 and 600 mg/dL) stimulate HSCs to proliferate and express type I collagen to a greater extent than normal serum concentrations of glucose (100 mg/dL) and that high glucose concentrations induce the growth of HSCs via MAP kinase pathways, which are activated by ROS produced by the NADPH oxidase system under the regulation of protein kinase C [6].

It is now widely accepted that ROS play a critical role in the development of hepatic fibrosis by increasing the deposition of ECM [7,8]. Various studies have demonstrated that an increase in ROS by a hepatic injury induces proinflammatory cytokines, such as tumor necrosis factor-α, transforming growth factor-β (TGF-β), interleukin-1β, and interleukin-6, which are critical for HSC activation and perpetuation [5]. Among these, TGF-β is thought to be an important cytokine in regulating the production, degradation, and accumulation of ECM proteins. 

*Ecklonia cava*, which is abundantly produced on Juju Island in Korea, is popular in Japan and Korea, where this valuable brown algae is utilized as an ingredient for food, animal feed, fertilizers and medicine. Polyphenolic compounds are in the blown algae are called as phlorotannins. Phlorotannin components, which are oligomeric polyphenols composed of phloroglucinol units, are responsible for the pharmacological activities of *Ecklonia cava*, and phlorotannins, such as eckol (a closed-chain trimer of phloroglucinol), 6,6'-bieckol (a hexamer) and phlorofucofuroeckol (a pentamer), were identified in the *Ecklonia cava* species [9]. Many researchers have reported that the *Ecklonia* species exhibits radical scavenging [10,11], anti-plasmin inhibiting [12,13], anti-mutagenic [14], bactericidal [15], HIV-1 reverse transcriptase and protease inhibition [16] and tyrosinase inhibitory [17] activities. It has also been reported that the amount of total polyphenolic compounds in *Ecklonia cava* is greater than that in other brown seaweeds [18]. 

In the present study, we examined the influence of *Ecklonia cava* extract (ECE) on high glucose-induced HSC activation and found that ECE suppressed the activation by decreasing the production of ROS and TGF-β.

## 2. Results

### 2.1. Changes in α-Smooth Muscle Actin Expression and Cell Proliferation after Hepatic Stellate Cell (HSC) Isolation

**Figure 1:**
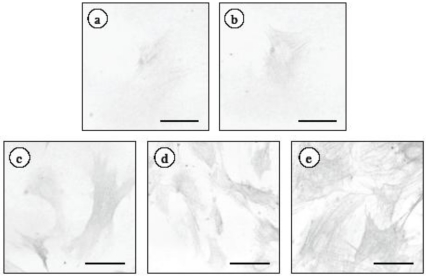
Time-dependent effect of α-SMA expression in HSCs. HSCs were cultured for 3, 5, 7, 9, 11 or 13 days. At the end of the incubation, cells were fixed with 4% paraformaldehyde overnight at 4 °C. An anti-α-SMA monoclonal antibody was used as the primary antibody. HSCs were cultured for (**a**) 3 days; (**b**) 5 days; (**c**) 7 days; (**d**) 9 days or (**e**) 13 days. Scale bars, 20 μm.

It is known that HSCs spontaneously demonstrate an activated phenotype and begin to proliferate after being plated. To ascertain when the phenotypical activation and proliferation of the HSCs are induced in our experimental conditions, we cultured HSCs for 13 days. The cell number was significantly increased after 7 days of culturing (Table 1). Similarly, the expression of α-smooth muscle actin (α-SMA), the most reliable marker for HSC activation, was observed in control HSCs after 7 days (Figure 1). The staining for α-SMA, using a computer with NIH image, were 15.7 ± 9.1, 16.1 ± 2.9, 22.4 ± 8.7, 37.6 ± 9.1 and 53.5 ± 6.7 pixels at 3, 5, 7, 9, 11 and 13 days after HSC isolation, respectively. Based on cell proliferation and the expression of α-SMA, HSCs that were cultured for 5 days were quiescent.

**Table 1:** Time-dependent effect of HSC proliferation.

Days after HSC isolation	Cell number (cells/field)
3	40.2 ± 8.2 ^a^
5	56.3 ± 7.1 ^a^
7	77.5 ± 7.8 ^b^
9	119.9 ± 20.9 ^c^
11	151.8 ± 18.8 ^d^
13	166.5 ± 13.9 ^d^

HSCs cultured for 3, 5, 7, 9, 11 or 13 days were counterstained with hematoxylin. Data are presented as means ± SD. Means with different superscript letters are significantly different (*p* < 0.01). Five independent experiments were performed.

### 2.2. Effect of ECE on High Glucose-Induced HSC Activation

**Figure 2:**
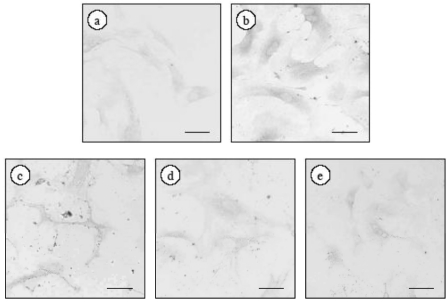
The effect of ECE on the high glucose-induced increase in the expression of type I collagen in HSCs. HSCs were incubated for 24 h with 400 mg/dL glucose. ECE (6.25, 12.5 or 25 μg/mL) was added simultaneously with the glucose. At the end of the incubation, cells were fixed with 4% paraformaldehyde overnight at 4 °C. An anti-type I collagen polyclonal antibody was used as the primary antibody. (**a**) Control (100 mg/dL glucose); (**b**) 400 mg/dL glucose; (**c**) 400 mg/dL glucose plus 6.25 μg/mL ECE; (**d**) 400 mg/dL glucose plus 12.5 μg/mL ECE; and (**e**) 400 mg/dL glucose plus 25 μg/mL ECE. Scale bars, 20 μm.

**Figure 3:**
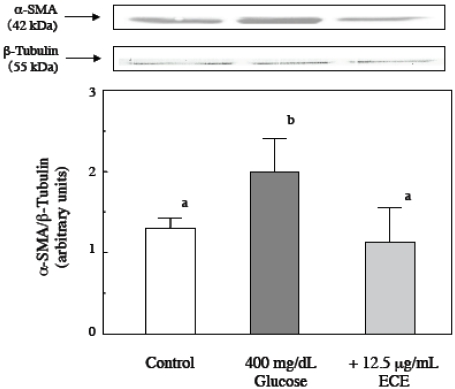
The effect of ECE on the high glucose-induced increase in the expression of α*-*SMA in HSCs. HSCs were treated with 400 mg/dL glucose for 24 h. ECE (12.5 μg/mL) was added simultaneously with the glucose. Cell lysis and western blot analysis were performed as described in the Materials and Methods section. Data shown are representative of three independent experiments.

In our previous study, we found that high glucose levels induced the activation of quiescent HSCs that were cultured for 5 days. To investigate the effect of ECE on high glucose-induced HSC activation, we examined the effect of ECE on the high glucose-induced increase in the expression of type I collagen in HSCs. HSCs were incubated for 24 h with 400 mg/dL glucose and various concentrations of ECE. As shown in Figure 2, treatment with ECE suppressed the high glucose-induced increase in the expression of type I collagen in HSCs. The staining for type I collagen, using a computer with NIH image, were 10.6 ± 3.8, 45.7 ± 5.7, 24.4 ± 7.1, 13.4 ± 3.4 and 14.9 ± 8.1 pixels, in the cells for control (100 mg/dL glucose), 400 mg/dL glucose, 400 mg/dL glucose plus 6.25 μg/mL ECE, 400 mg/dL glucose plus 12.5 μg/mL ECE and 400 mg/dL glucose plus 25 μg/mL ECE, respectively. The most effective concentration by immunostaining was found to be 12.5 µg/mL ECE, and this concentration was used for the following experiments. To quantitatively detect the expression of type I collagen, we measured the levels of intracellular type I collagen by Western blot analysis and showed that treatment of HSCs with 12.5 μg/mL ECE for 24 h markedly suppressed the high glucose-induced increase in the expression of type I collagen (Figure 3).

### 2.3. Effect of ECE on the Proliferation of High Glucose-Treated HSCs

**Figure 4:**
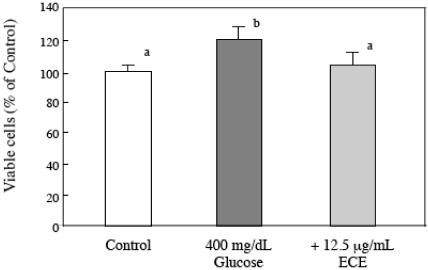
The effect of *Ecklonia cava* extract (ECE) on the proliferation of high glucose-treated HSCs. HSCs were treated with 400 mg/dL glucose for 24 h. ECE (12.5 μg/mL) was added simultaneously with the glucose. Cell viability was measured using an MTT assay. Each value is the mean ± SD. Values without a common letter are significantly different (*p* < 0.01). Five independent experiments were performed.

To investigate the effect of ECE on high glucose-induced HSC activation, we also examined the effect of ECE on the high glucose-induced increase in the proliferation of HSCs. HSCs were incubated for 24 h with 400 mg/dL glucose and 12.5 μg/mL ECE. As shown in Figure 4, treatment with ECE reduced the high glucose-induced cell proliferation to the level of the control cells.

### 2.4. Effect of ECE on Intracellular ROS Levels of High Glucose-Treated HSCs

**Figure 5:**
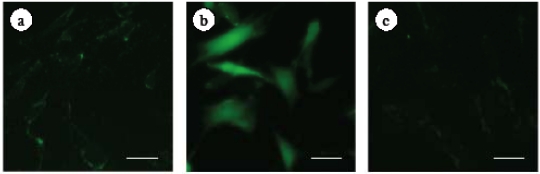
The effect of ECE on the intracellular ROS levels of high glucose-treated HSCs. HSCs were incubated for 2 h with 400 mg/dL glucose. ECE (12.5 μg/mL) was added simultaneously with the glucose. During the last 30 minutes of culture, the cells were incubated with 2.4 mM DCFH-DA. (**a**) Control (100 mg/dL glucose); (**b**) 400 mg/dL glucose; and (**c**) 400 mg/dL glucose plus 12.5 μg/mL ECE. The data shown are representative of three independent experiments. Scale bars, 20 μm.

To investigate the relationship between high glucose-induced HSC activation and ROS formation, we measured the level of intracellular ROS using 2',7'-dichlorodihydrofluorescein diacetate (DCFH-DA), which is converted to the highly fluorescent DCF in the presence of intracellular ROS. The increase in ROS formation in high glucose-treated HSCs was inhibited by the addition of ECE (Figure 5). 

### 2.5. Effect of ECE on the Intracellular MDA Levels of Glucose-Treated HSCs

**Figure 6:**
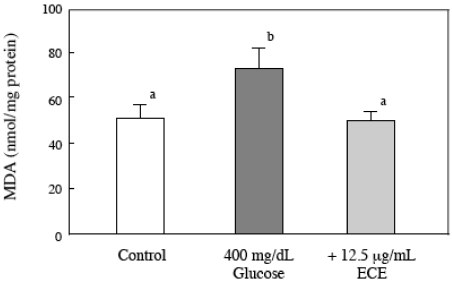
Effect of ECE on MDA levels in high glucose-treated HSCs. HSCs were treated with 400 mg/dL glucose for 6 h. ECE (12.5 μg/mL) was added simultaneously with the glucose. After incubation, the intracellular MDA levels were assayed using a TBARS assay, as described in the Materials and Methods section. Data are presented as means ± SD. Values without a common letter are significantly different (*p* < 0.01). Five independent experiments were performed.

We examined the effect of ECE on the high glucose-induced increase of intracellular lipid peroxidation using a TBARS assay. HSCs were incubated for 6 h with 400 mg/dL glucose and 12.5 μg/mL of ECE. The ECE treatment returned the intracellular MDA level to the level of the control cells (Figure 6).

### 2.6. Effect of ECE on the Intracellular GSH Levels of High Glucose-Treated HSCs

**Figure 7:**
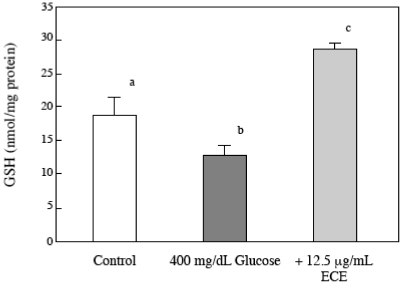
The effect of ECE on the intracellular GSH levels of high glucose-treated HSCs. HSCs were treated with 400 mg/dL glucose for 2 h. ECE (12.5 μg/mL) was added simultaneously with the glucose. After the incubation, the intracellular GSH levels were assayed by HPLC, as described in the Materials and Methods section. Data are presented as means ± SD. Values without a common letter are significantly different (*p* < 0.01). Five independent experiments were performed.

To investigate the relationship between intracellular GSH levels and high glucose-induced HSC activation, we measured the intracellular GSH levels in HSCs using HPLC. As shown in Figure 7, the levels of intracellular GSH 2 h after a high glucose treatment were lower than those of the control cells. However, ECE not only reversed the decrease in GSH but also induced higher GSH levels than those in control cells.

### 2.7. Effect of ECE on Bioactive TGF-β1 in High Glucose-Treated HSCs

**Figure 8:**
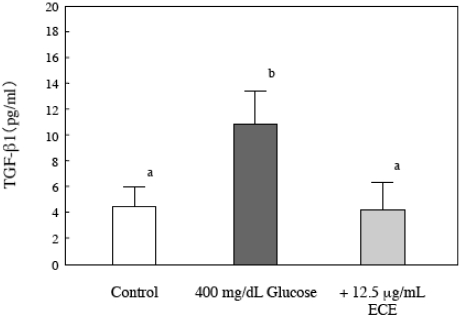
The effect of ECE on the secretion of bioactive TGF-β1 by high glucose-treated HSCs. HSCs were treated with 400 mg/dL glucose for 24 h. ECE (12.5 μg/mL) was added simultaneously with the glucose. After the incubation, the levels of secreted bioactive TGF-β1 were determined by an ELISA, as described in the Materials and Methods section. Data are presented as means ± SD. Values without a common letter are significantly different (*p* < 0.01). Five independent experiments were performed.

TGF-β1 is a potent profibrogenic signaling factor that triggers the expression, accumulation and deposition of collagen [19,20]. Therefore, we measured the levels of TGF-β1 in the cell culture medium using a Quantikine TGF-β1 ELISA kit (R&D Systems). As shown in Figure 8, a high glucose concentration (400 mg/dL) stimulated the cells to secrete bioactive TGF-β1, and the stimulation was suppressed upon treatment of the HSCs with ECE.

## 3. Discussion

The significant finding of this study is that ECE suppressed the high glucose-induced HSC activation *in vitro*. To the best of our knowledge, the involvement of ROS and/or GSH inhibition and the downregulation of TGF-β secretion by ECE are novel. 

Nonalcoholic fatty liver disease (NAFLD) is a common chronic liver disease that ranges in severity from steatosis to cirrhosis. The prevalence of NAFLD has been estimated to be between 20% and 30% in the general population, but this value is much higher (~70–80%) in patients with type 2 diabetes mellitus, who are also at a higher risk of developing advanced fibrosis and cirrhosis [21]. In addition, Picardi *et al*. showed that hyperglycemia and hyperinsulinemia might accelerate liver fibrosis and cirrhosis [3]. Nan *et al*. have shown that rosiglitazone, which is used as a clinical treatment for insulin resistance in patients with type 2 diabetes mellitus, ameliorated hepatic fibrosis by activating PPARγ, which can inhibit HSC activation and suppress the expression of TGF-β1 and connective tissue growth factor. When the drug was given to mice fed a high-fat, methionine-choline deficient diet for 8 weeks to induce hepatic fibrosis, it decreased the severity of the liver disease [22]. Currently, no agents or natural products have been confirmed as preventing the activation of HSCs in diabetic liver fibrosis. In this study, we examined the effect of ECE on high glucose-induced HSC activation.

In cirrhotic livers, HSCs are responsible for the increased production and deposition of the ECM. Activation of HSCs *in vivo* and *in vitro* includes the increased expression of type I collagen, the expression of cytoskeleton markers such as α-SMA, and increased proliferation [23,24]. Sugimoto *et al*. have shown that high glucose concentrations stimulated the cell proliferation of HSCs and the production of type I collagen by HSCs. However, the mechanisms of hepatic fibrosis, which is promoted by high glucose concentrations, are not well defined [6]. We confirmed that high glucose concentrations (400 and 600 mg/dL) stimulated cell proliferation and type I collagen expression by HSCs to a greater extent than normal serum glucose concentrations (100 mg/dL) (data not shown). In this study, we showed that the secretion of TGF-β1 and the formation of ROS were stimulated and that the intracellular GSH levels were decreased in HSCs at high glucose concentrations.

ROS has been thought to be an important trigger for HSC activation and for promoting the expressions of fibrogenic molecules such as α-SMA, TGF-β, and type I collagen. TGF-β plays a critical role in the development of hepatic fibrosis through its stimulating effect on matrix protein generation and its inhibitory effect on matrix protein removal [20,25]. The expression of TGF-β is increased in various models of human liver disease, which range from cholestatic liver disease and hepatitis to liver cirrhosis [26,27,28]. Recent studies have demonstrated that TGF-β is a potent inducer of both the α-1(I) and α-2(I) collagen genes. In addition, a TGF-β-responsive element has been mapped to the promoter region of the α-2(I) collagen gene [29]. The finding of this study that a high glucose concentration intensified the generation of ROS, which was then followed by HSC activation in association with enhanced TGF-β secretion, adds new information to the mechanism of liver fibrogenesis. The increases in ROS formation and TGF-β secretion in high glucose-induced HSCs were inhibited by the addition of ECE. 

The levels of ROS are modulated not only by the amount of ROS produced but also by antioxidant levels. The production of ROS leads to antioxidant depletion, thereby amplifying the biological consequences of oxidant stress. GSH has been implicated in various cellular events, such as the inflammatory response, regulation of cell proliferation, modulation of redox-regulated signal transduction and remodeling of the extracellular matrix [30]. In this experiment, we found that the level of intracellular GSH was decreased after treatment with high glucose when compared to that of the control cells. The decrease in GSH levels in high glucose-treated HSCs was suppressed by treatment with ECE. ECE not only reversed the decrease in GSH but also induced greater GSH levels than those in control cells. Kang *et al*. have shown that phloroglucinol, a phlorotannin compound isolated from *Ecklonia cava*, restored the level of GSH and the protein expression of the catalytically active subunit glutamate-cysteine ligase, which is a rate-limiting enzyme in GSH biosynthesis, in cells exposed to γ-rays [31].

Polyphenolic compounds are abundant in seaweeds, and the polyphenolic compounds that are contained in brown algae are called phlorotannins. *Ecklonia cava*, which is a brown algae, has various phlorotannins that are highly hydrophilic compounds [32]. Ahn *et al*. investigated the potential antioxidant activities of three phlorotannins (phloroglucinol, eckol and dieckol) purified from *Ecklonia cava* and indicated that those phlorotannins showed notable radical scavenging activities; in particular, eckol had a superior scavenging activity for free radicals and inhibited DNA damage [33]. 

These studies suggested that ECE is useful for preventing the development of diabetic liver fibrosis. However, further studies are required to ascertain whether ECE may be beneficial in high glucose-induced or NASH-associated hepatic fibrosis in animals.

## 4. Materials and Methods

### 4.1. Materials

Nycodenz was obtained from Nycomed Pharma AS (Oslo, Norway). Pronase E was purchased from Merck (Darmstadt, Germany). Deoxyribonuclease I was obtained from Roche LTD (Basel, Switzerland). Collagenase was purchased from Wako Pure Chemical Co., Ltd. (Osaka, Japan). DMEM was obtained from Nissui Pharmaceutical Co. Ltd. (Tokyo, Japan). Fetal bovine serum (FBS) was purchased from Nichirei Biosciences, Inc. (Tokyo, Japan). Monoclonal mouse anti-human smooth muscle actin antibody 1A4, biotinylated goat anti-mouse immunoglobulin and biotinylated goat anti-rabbit immunoglobulin and horseradish peroxidase-labeled streptavidin-biotin complex were obtained from DAKO A/S (Glostrup, Denmark). Rabbit anti-rat collagen type I polyclonal antibody was obtained from Chemicon International, Inc. (Temecula, CA, USA). The Quantikine TGF-β1 ELISA kit was obtained from R&D Systems, Inc. (Minneapolis, Minnesota, MN, USA). Other chemicals used in this study were special-grade commercial products purchased from WAKO Pure Chemical Co., Ltd. (Osaka, Japan).

### 4.2. *Ecklonia cava* Extract

A commercially available polyphenol extract from *Ecklonia cava* (Seapolynol, Livechem Inc, Jeju, Korea) was used. The total polyphenol content of the *Ecklonia cava* extract was 99.4%, as measured by the Folin-Ciocalteu reagent using phloroglucinol as a standard. Notable compounds in the *Ecklonia cava* extract that were identified by HPLC were dieckol (8.2%), 8,8'-bieckol (2.8%), 2-*O*-(2,4,6-trihydroxyphenyl)-6,6'-bieckol (2.1%), 6,6'-bieckol (1.5%), phlorofurofucoeckol-A (1.4%), eckol (0.6%), 2-phloroeckol (0.4%), 7-phloroeckol (0.4%) and phlorotannin A (0.4%) (Waters, column: CAPCELL PAK ODS column (4.6 × 250 mm); eluent: 30% aqueous MeOH; flow rate: 0.8 mL/min) [11].

### 4.3. Animals

Male Wistar rats, which weighed 300–350 g, were purchased from Japan SLC Inc. (Shizuoka, Japan) and housed at constant temperature with free access to water and standard rat chow (Labo MR stock; Japan SLC, Inc., Shizuoka, Japan). Animal experiments followed our institution’s criteria for the care and use of laboratory animals in research, which were in accordance with the guidelines for animal experimentation at Osaka City University.

### 4.4. Isolation and Culturing of HSCs

HSCs were isolated from male Wistar rats as previously described [34]. HSCs were identified by their typical star-like configuration and a vitamin A autofluorescence. The purity was always higher than 95%. In the experiments to ascertain changes in α-smooth muscle actin (α-SMA) expression and cell proliferation after HSC isolation, the cells were plated at 5 × 10^5^ cells/mL on uncoated culture dishes in 1.5 mL of DMEM containing 10% FBS and supplemented with antibiotics (10^5^ U/L of penicillin G and 500 mg/L of streptomycin) until 13 days. The medium was changed to fresh DMEM containing 10% FBS every 2 days. For the experiments on high glucose-induced HSC activation, the cells were plated at 5 × 10^5^ cells/mL on uncoated culture dishes in 1.5 mL of DMEM containing 10% FBS and supplemented with antibiotics for 2 days and then cultured in fresh medium without serum for 24 h. After a pre-incubation, the cells were cultured in DMEM with different concentrations of glucose.

### 4.5. Immunohistochemistry

At the end of the incubation, the HSCs were fixed with 4% paraformaldehyde overnight at 4 °C. We used either an anti-α-SMA monoclonal antibody (1:100 dilution) or an anti-type I collagen polyclonal antibody (1:200 dilution) as the primary antibody. The samples were sequentially incubated with 0.3% hydrogen peroxide, followed by normal goat serum, then with the primary antibody for 1 h at room temperature, and afterwards with the biotinylated anti-mouse or anti-rabbit goat immunoglobulins for 30 min. This was then followed by an incubation with the horseradish peroxidase-labeled streptavidin-biotin complex for 30 min. For the peroxidase reaction, 3,3'-diaminobenzidine tetrahydrochloride (DAB) with a nickel chloride color modification was incubated for 5 min until the desired color intensity had developed. Quantification of the intensity of α-SMA and type I collagen expression was analyzed using a computer with the NIH image version 1.62 (National Institutes of Health, Bethesda, MD, USA). 

### 4.6. Western Blot Analysis of α-SMA

Cells were harvested, washed twice with cold PBS and then dissolved with lysis buffer X (10 mM HEPES (pH 7.6), 10 mM KCl, 0.1 mM EDTA, 0.5% Nonidet P40, 1 mM dithiothreitol, 0.5 mM phenylmethylsulfonyl fluoride). After freezing and thawing twice with liquid nitrogen, the cells were sonicated and centrifuged at 800 × g for 10 min at 4 °C. The supernatant was collected for the cytosolic fraction. The precipitant was dissolved with lysis buffer Y (150 mM NaCl, 50 mM tris (pH 7.2), 1 mM EDTA, 1% Nonidet P40, 10 µg/mL leupeptin, 10 µg/mL pepstatin A and 100 µg/mL phenylmethylsulfonyl fluoride) for 30 min. Finally, the solution was sonicated and centrifuged at 2000 × g for 20 min at 4 °C, and the supernatant was collected for the nuclear fraction. Equal amounts of protein were loaded into the lanes of 10% SDS-PAGE gels, and the separated proteins were blotted to 0.45 µm polyvinylidene difluoride (PVDF) membranes (Amersham Pharmacia Biotech, Inc., Uppsala, Sweden). After blocking overnight with 0.1% Tween-20 and 5% non-fat dry milk in TBS, the membrane was incubated with an anti-α-SMA antibody for 1 h at room temperature. After washing, the membrane was re-incubated with biotinylated anti-mouse goat immunoglobulin (diluted 1:1000) for 1 h at room temperature. Next, the membrane was washed several times and then incubated with horseradish peroxidase-coupled streptavidin (diluted 1:200) for 1 h at room temperature. After several washing steps, the color reaction was developed with DAB. Densitometric analysis of the protein bands was performed using the software Scion Image (Scion Corporation, Frederick, MD, USA).

### 4.7. Determination of Intracellular GSH Levels

The intracellular GSH levels were determined according to the method of Sack *et al*. [35]. Cells were collected with Tris-HCl buffer (25 mM Tris adjusted to pH 7.5 with HCl). After sonication and centrifugation, the supernatant was used for an HPLC assay. For assaying GSH, 300 µL of supernatant was mixed with 300 µL of borate buffer (0.56 N boric acid adjusted to pH 10.4 with NaOH) and 50 µL of an OPA solution (10 mg/mL OPA in 10% methanol). GSH was separated on an ODS-II column (4.6 × 150 mm; particle size 5 µm; Shimadzu Techno-Research, Kyoto, Japan) using solvents A (30 mM sodium acetate adjusted to pH 6.0 with acetic acid) and B (92.3% methanol/7.7% acetonitrile, v/v). The sample was eluted with 96% solvent A and 4% solvent B and then with a programmed solvent gradient using a linear gradient curve. The gradient changed from 4 to 10% of solvent B from 0 to 5 min, from 10 to 14.9% of solvent B from 5 to 15 min, and from 14.9 to 4% of solvent B from 20 to 21 min at a flow rate of 0.45 mL/min. The fluorescence of the eluted fractions was measured using an RF1520 fluorescence monitor at excitation and emission wavelengths of 230 and 445 nm, respectively, to assay the GSH levels (JASCO Corporation, Tokyo, Japan). 

### 4.8. MTT Assay

The MTT [3(4,5-dimethylthiazol-2-yl)-2,5-diphenyltetrazolium-bromide] assay, an index of cell viability and growth, is based on the ability of viable cells to reduce a yellow water-soluble dye to a dark-blue insoluble formazan product. After 24 h of culture, the cells were incubated with the MTT solution for 2 h. The cells showing an MTT reaction were quantified at 600 nm using a multilabel counter (Wallac 1420 ARVOsx, Perkin Elmer Inc., Waltham, MA, USA). Cell survival was estimated as a percentage of the value of the untreated controls.

### 4.9. Intracellular ROS Formation

A relatively specific probe for the presence of hydrogen peroxide, 2',7'-dichlorodihydrofluorescein diacetate (DCFH-DA), was used to analyze the intracellular ROS formation [36,37]. Cells were incubated with 2.4 mM DCFH-DA (5 µL) for the last 30 min of the glucose treatment. Cells were washed with PBS twice and resuspended in Hank’s solution. The fluorescence intensities were measured using a multilabel counter (Wallac 1420 ARVOsx, Perkin Elmer Inc.) with an excitation wavelength of 485 nm and an emission wavelength of 535 nm. The amount of intracellular ROS was calculated from a standard curve derived from 2',7'-dichlorofluorescein (DCF). The protein concentration was measured using the Bradford method [38]. For visualization of the intracellular fluorescence, the cells were observed with an FSX100 Bio Imaging Navigator, which is an all-in-one fluorescence imaging system (Olympus Corporation, Tokyo, Japan).

### 4.10. Evaluation of Intracellular Lipid Peroxidation

Lipid peroxidation was assessed by determining the rate of production of thiobarbituric acid (TBA)-reactive components, which mainly detect malonaldehyde (MDA) [39]. After incubating for 6 h, the HSCs were collected and resuspended in 500 µL of Hank’s solution. After freezing and thawing, the 250 µL suspension was diluted to 500 µL with distilled water and then mixed with 15 µL of 50 mM butylated hydroxytoluene (BHT) and 1 mL of the thiobarbituric acid (TBA) solution. The mixtures were heated at 95 °C for 15 min. The fluorescence intensities were measured using a multilabel counter (Wallac 1420ArVOsx, Perkin Elmer Inc.) with an excitation wavelength of 485 nm and an emission wavelength of 535 nm. Tetramethoxypropane was used for a standard, and the results are expressed as nmol equivalents of MDA.

### 4.11. Measurement of Activated TGF-β1 Concentrations

TGF-β1 was analyzed using a Quantikine TGF-β1 ELISA kit (R&D Systems, Inc.) according to the manufacturer’s instructions. The assay employs the quantitative sandwich enzyme immunoassay technique. The intensity of the color was measured at 450 nm by a multilabel counter (Wallac 1420 ARVOsx, Perkin Elmer Inc.). The concentrations of the total TGF-β1 was calculated from a standard curve derived from various defined concentrations of recombinant TGF-β1. 

### 4.12. Statistical Analysis

Results are presented as the means ± S.D. Statistical comparisons were performed between groups by a one-way analysis of variance and a *post-hoc* multiple comparison using a Tukey’s test. A *p*-value less than 0.05 was considered significant.

## 5. Conclusions

In conclusion, the results obtained in this study indicate that high glucose concentrations trigger an increase in collagen synthesis by HSCs, and this may be related to or involved in an increased formation of ROS, a depletion of the intracellular GSH content and an increase in the expression of TGF-β1. The suppression of the high glucose-induced HSC activation by ECE was mediated by an inhibition of the ROS and/or GSH signal and a downregulation of TGF-β1 secretion. ECE may be useful in preventing the development of diabetic liver cirrhosis.
